# Variance Analysis of genes in bacteria producing the enzyme chlorohydrolase atrazine used in the bioremediation of xenobiotics

**DOI:** 10.1186/1753-6561-8-S4-P195

**Published:** 2014-10-01

**Authors:** Alisson Silva, Rodrigo Pereira, Maricy Bonfá

**Affiliations:** 1 Federal University of Grande Dourados, Dourados, MS, Brazil

## 

Bioremediation is the process in which are employed organisms, plants or microorganisms for removal or reduction of pollutants into the environment, either on the ground or water. The biological process of bioremediation is ecologically more suitable and effective for treatment of contaminated environments contaminated with organic and recalcitrant molecules and toxic metals. Recalcitrant molecules which pollute the environment are generally called xenobiotic are the result of anthropic action, as they are synthetic chemical compounds produced industrially by humans and include plastics, solvents, lubricants, detergents, pesticides explosive, comprising thus a large number of molecules of different applications, potential toxicity, and remain in the environment. When these elements are in the natural ecosystem in large quantities, exhibit toxic effects or other undesirable effects by organisms [1,2]. Atrazine pertecente the class of *s-*triazines is considered a xenobiotic being widely used in agriculture as a herbicide and thus widely detected in groundwater as far surface [3]^4^. The key enzyme for the initial biodegradation of this compound is the atrazine chlorohydrolase (*Atza*), performs an action of hydrolytic removal of the ring of the compound [3]. Currently, through the use of bioinformatics tools can be studied in depth the composition and organization of microorganisms and their genetic variations. The NCBI database provides many information about the organisms, such as sequences of specific genes or entire genomes of organisms ^5^. This study aimed to evaluate the variations in the gene that codes for the enzyme atrazine chlorohydrolase in 10 bacterial species through the construction of a phylogenetic tree. Through observing the results could be verified that among analyzed bacteria only *Rhodococcus corallinus *emerges from a different ancestral in the phylogenetic tree and the remainder strains analyzed constitute a group rate descendant of a single common ancestral, with slight variation in the gene encoding the enzyme atrazine chlorohydrolase.
